# Application of artificial intelligence in risk assessment and management of venous thromboembolism: scoping review

**DOI:** 10.3389/fphys.2025.1664470

**Published:** 2025-10-08

**Authors:** Yujia Gu, Yang Yang, Xiaojie Gao, Yanru Wang, Luo Yang, Yehong Wei

**Affiliations:** ^1^ College of Nursing, Zhejiang Chinese Medical University, Hangzhou, China; ^2^ College of Continuing Education, Bengbu Medical University, Anhui, China; ^3^ Department of Intensive Care, The Second Affiliated Hospital of Zhejiang Chinese Medical University, Hangzhou, China

**Keywords:** artificial intelligence, AI models, venous thromboembolism, scoping review, VTE (venous thromboembolism)

## Abstract

**Objective:**

To systematically analyze the current application status of artificial intelligence (AI) in risk assessment and management of venous thromboembolism (VTE), evaluate the predictive performance of AI models and identify key risk factors, thereby providing evidence-based references for optimizing clinical VTE prevention and treatment strategies.

**Methods:**

A scoping review framework was used. We searched for literature in both Chinese (CNKI, Wanfang, CBM) and English databases (PubMed, Web of Science, Embase, CINAHL, and The Cochrane Library) to find studies on AI applications in VTE risk assessment, covering the time from when the databases started until 10 March 2025. By creating research questions, reviewing the literature, gathering data, and summarizing the results, we organized various AI models, assessed how accurately they predicted outcomes, and looked at important risk factors.

**Results:**

This review included a total of 23 studies. AI models showed better accuracy in predicting VTE risk, with AUC values between 0.740 and 0.990, greatly surpassing traditional scoring tools. Key risk factors identified included patient-related factors, disease-related factors, treatment-related factors, laboratory indicators, and catheter-related factors.

**Conclusion:**

AI technology shows remarkable advantages in VTE risk assessment by integrating multi-source data to achieve dynamic and personalized prediction. Future research should aim to conduct studies across multiple centers to confirm how useful these models are in real-life situations and also look into combining real-time monitoring data with AI to enhance the accuracy of preventing and treating VTE, which will help lower the number of cases and improve patient results.

## 1 Introduction

Venous Thromboembolism (VTE), which includes deep vein thrombosis (DVT) and pulmonary embolism (PE), is a prevalent vascular disorder that is associated with significant morbidity, mortality, and healthcare burden (Henke et al., 2020). Globally, approximately 10 million cases of VTE occur annually (Global Burden of VTE, 2023), with a steadily rising incidence—particularly among hospitalized patients, surgical populations, and cancer patients, who face significantly elevated risks. As reported by the World Health Organization (WHO), VTE ranks as the third leading cause of cardiovascular mortality, claiming over 1 million lives yearly (Porfidia et al., 2020). Without timely intervention, VTE may progress to chronic complications such as post-thrombotic syndrome and chronic pulmonary hypertension, severely impairing patients’ quality of life (Zhai et al., 2019). Early detection, accurate risk prediction, and prompt intervention are thus critical to mitigating the disease burden. Current standard risk assessment tools, such as the Caprini and Padua scores, while valuable, exhibit several inherent limitations that constrain their predictive performance and clinical utility. These models predominantly rely on a limited set of static, clinically apparent variables, often captured at a single time point. This approach fails to capture the dynamic evolution of patient risk throughout the care continuum, integrates poorly with electronic health record (EHR) systems for real-time calculation, and cannot effectively synthesize complex, non-linear interactions among multifactorial risks or leverage unstructured data from clinical notes. Consequently, their predictive accuracy remains modest (AUC: 0.60–0.75), leading to both over-prophylaxis in low-risk patients and under-prophylaxis in high-risk individuals, which contributes to the persistent burden of VTE-related complications (Stevens et al., 2022).

Faced with these challenges, there is a pressing need to explore novel methodologies to overcome the limitations of traditional scoring tools. In this context, artificial Intelligence (AI) has emerged as a transformative technology with significant potential in medical risk assessment (Zhou, 2023; Haug and Drazen, 2023). In contrast to conventional scoring systems, AI leverages machine learning (ML) and deep learning (DL) algorithms to overcome these limitations, representing a paradigm shift in the field (Rajkomar et al., 2019). AI algorithms can dynamically process high-dimensional, multi-source data—including structured electronic health record (EHR) fields, longitudinal laboratory parameters, and unstructured imaging and clinical narratives—to identify complex, latent patterns, thereby enabling personalized and dynamic prediction. Currently, AI models such as random forests, gradient boosting decision trees, and neural networks have been preliminarily validated in predicting outcomes related to cardiovascular diseases, sepsis, and cancer. For example, AI-based tools have been successfully developed to predict stroke risk in patients with atrial fibrillation, major adverse cardiovascular events after acute coronary syndrome, and cancer-associated thrombosis events. These models generally demonstrate superior predictive performance (as measured by AUC values) compared to traditional clinical scoring rules (Deo, 2015; You et al., 2023; Gaviria-Valencia et al., 2023). The success of AI in these domains provides a robust methodological foundation and promising prospects for the application of AI in VTE risk assessment. As such, AI technology offers a novel pathway to overcome the limitations of traditional VTE evaluation. Nevertheless, the development of AI applications specifically for VTE risk assessment has progressed relatively slowly and faces unique challenges. The construction of powerful AI models is hindered by significant data-related obstacles, including heterogeneity in VTE data collection and annotation across institutions, the high-dimensional and often incomplete nature of real-world clinical datasets, and the inherent complexity in defining and labeling VTE outcomes for model training. Therefore, although existing AI research in VTE has primarily focused on diagnostic image analysis or treatment outcome prediction, efforts dedicated to developing and validating AI-driven risk assessment tools remain in their nascent stages (Yang et al., 2023; Vollmer et al., 2020).

This study aims to conduct a scoping review to systematically map the current landscape of AI applications in venous VTE risk assessment. Specifically, we will evaluate the predictive performance of emerging AI models in comparison to traditional risk scores, identify key predictive features utilized by these algorithms, and critically appraise the methodological rigor and clinical readiness of the existing evidence. Furthermore, this review will elucidate the transformative potential of AI, assess its advantages and challenges in VTE risk prediction, and provide guidance for future research. Ultimately, this work is expected to contribute to optimized VTE prevention and treatment strategies, reduce the incidence of VTE-related complications, alleviate the disease burden, and improve patient outcomes.

## 2 Materials and methods

### 2.1 Defining the research question

After preliminary literature screening, the research question was formulated as follows: The study aimed to conduct a scoping review on the application and management of artificial intelligence in risk assessment for venous thromboembolism (VTE) in both domestic and international contexts. The key components were defined using the PICO-S framework: P (Population): Patients with venous thromboembolism. I (Intervention): Risk assessment tools, including Random Forest models, XGBoost models, deep learning models, natural language processing (NLP), etc. C (Comparison): Traditional risk assessment scales such as the Caprini score and Padua score. O (Outcome): Outcome measures include the area under the curve (AUC), sensitivity, specificity, etc. S (Study design): Study types such as retrospective cohort studies, prospective cohort studies, randomized controlled trials (RCTs), systematic reviews, and meta-analyses.

### 2.2 Literature search

**FIGURE 1:**
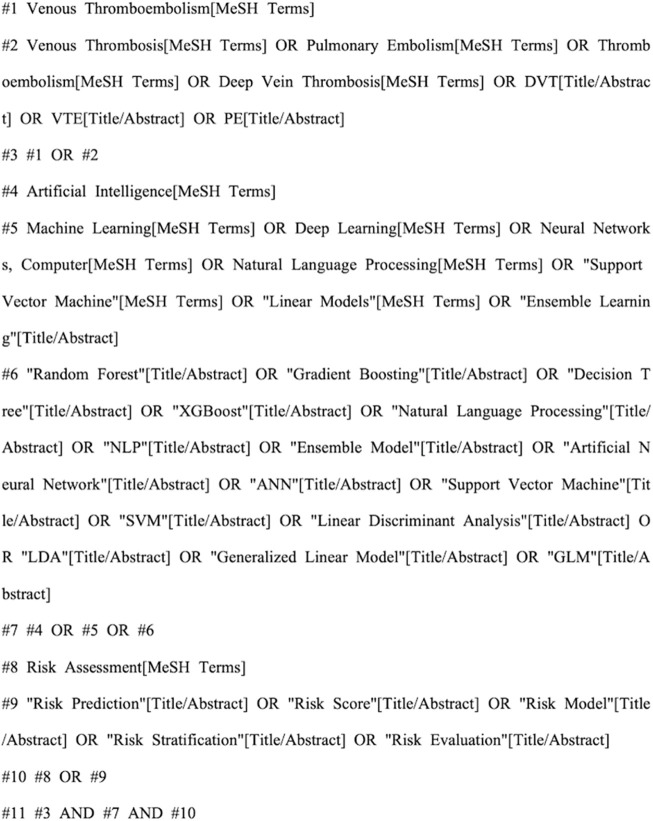
PubMed retrieval strategy.

A comprehensive search was conducted using a combination of subject headings and keywords in the following databases: CNKI, Wanfang, CBM, PubMed, Web of Science, Embase, CINAHL, and the Cochrane Library. The search encompassed all published studies on the application of artificial intelligence in venous thromboembolism (VTE) risk assessment, with a time frame from database inception to 10 March 2025. For Chinese databases (like CNKI), the search method was (Artificial Intelligence OR Large Language Model OR AI) AND (Venous Thromboembolism OR Deep Vein Thrombosis OR Pulmonary Thromboembolism OR VTE OR DVT OR PTE OR PE) AND (Risk Assessment OR Associated Risk OR Related Factors OR Risk Factors OR Predictive Factors). We used a combination of MeSH terms and free-text keywords for English databases like PubMed, to ensure comprehensive retrieval and precision of the literature, we supplemented our search by manually reviewing the reference lists of all included studies. Figure 1 presents the detailed search strategy.

### 2.3 Inclusion and exclusion criteria

#### 2.3.1 Literature inclusion criteria

(1) The objective was to explore the application of artificial intelligence in the risk assessment of venous thromboembolism; (2) The study population was VTE patients, aged ≥18 years; (3) There is no limit to the type of research; (4) Articles published in Chinese or English.

#### 2.3.2 Literature exclusion criteria

(1) Duplicate publications; (2) Documents with incomplete data or unable to obtain the full text; (3) The language of the document is not Chinese or English; (4) Non venous thrombosis-related literature.

### 2.4 Literature extraction

EndNote20.0 software was used to eliminate the duplicate of the imported literature, Prior to the formal screening process, a standardized data extraction form was developed based on the predetermined inclusion and exclusion criteria. To ensure accuracy and consistency in the extraction process, a pilot test was conducted using this form. Two researchers independently performed trial extractions on five randomly selected articles. Their results were then compared, and any discrepancies in the understanding or application of the extraction items were discussed. Through iterative calibration of the definitions and criteria, a consensus was reached, resulting in a finalized version of the standardized form for use in the formal data extraction. After the first round of title and abstract screening, the literature that did not meet the inclusion criteria was excluded, exclusion criteria included mismatches between the research focus and the study’s subject matter, incomplete data, and unavailability of full-text articles. Two researchers independently screened and extracted the remaining literature using two rounds of full-text reading, based on the inclusion and exclusion criteria: The first phase focused on capturing general study characteristics, including the first author, publication year, country of origin, study design, type of AI model utilized, study population, and sample size. The second phase involved extracting key data directly relevant to the research objectives, which encompassed model performance metrics on the test/validation sets—such as the area under the curve (AUC) with 95% confidence intervals, sensitivity, and specificity—as well as significant VTE risk factors identified in the studies. At the final full-text screening stage for inclusion, inter-rater reliability was assessed, yielding a Cohen’s Kappa coefficient of 0.84, which indicates an almost perfect level of agreement between the two independent researchers. If the two researchers disagree, the third researcher will arbitrate, and a consensus will be reached after the third round of discussion. Finally, the data of the included literatures were summarized by standardized tables (see Table 1), and the extracted contents include basic information: author, publication year, tool type, and artificial intelligence technology; Clinical characteristics: application population and main evaluation contents; Model effectiveness: test set AUC, validation set AUC, sensitivity, and specificity.

**TABLE 1:** Evaluation of risk factors and reliability and validity indicators of included literature.

Developer	Tool type	AI technology	Clinical application population	Main assessment contents	Test set AUC*	Validation set AUC#	Sensitivity	Specificity
Wang et al. (2020)	Prediction model	Random forest model	376 inpatients	1, 2, 3, 4, 5, 6, 7, 8, 9, 10, 11	0.856 (95% CI: 0.807–0.905)	0.782 (95% CI: 0.807–0.905)	0.733* 0.595#	0.800* 0.865#
Nudel et al. (2021)	Prediction model	Gradient lifting decision tree	436,807 patients undergoing bariatric surgery	1, 2, 3, 6, 12, 13, 14	0.670 (95% CI: 0.64–0.69)	0.660 (95% CI: 0.64–0.69)	0.711* 0.595#	0.722* 0.832#
高远 and 李建涛 (2021)	Prediction model	Random forest model	15,856 orthopedic patients	4, 14, 15, 16	0.890	-	0.290*	0.970*
Wang et al. (2021)	Prediction model	natural language processing	51,383 inpatients	2, 12, 16	0.970 (95% CI: 0.95–0.98)	0.980 (95% CI: 0.97–0.99)	0.890* 0.870#	0.970* 0.960#
Liu et al. (2021)	Prediction model	Support vector machine model	573 inpatients	4, 16, 17	0.904 (95% CI: 0.87–0.94)	0.875 (95% CI: 0.804–0.944)	0.590* 0.590#	0.990* 0.990#
Ryan et al. (2021)	Prediction model	Gradient lifting decision tree	99,237 inpatients	4, 6, 16	0.830 (95% CI: 0.81–0.85)	0.850 (95% CI: 0.83–0.87)	0.800* 0.800#	0.660* 0.750#
Park et al. (2021)	Prediction model	Random forest model	92,481 inpatients	2, 6, 10, 14, 18, 19, 20	0.840	-	0.740*	0.800*
Jin et al. (2022)	Prediction model	Linear discriminant analysis	231 inpatients	2, 4, 16, 18, 20	0.773 (95% CI: 0.722–0.818)	-	0.754*	0.660*
Li et al. (2022)	Prediction model	Generalized linear model	338 inpatients	2, 16	0.839 (95% CI: 0.794–0.884)	0.826 (95% CI: 0.755–0.897)	0.733* 0.708#	0.828* 0.863#
Wang et al. (2023a)	Prediction model	Integrated model^①^	6,987 inpatients	2, 3, 4, 6, 10, 12, 14, 16	0.920 (95% CI: 0.895–0.936)	0.920 (95% CI: 0.895–0.936)	0.802* 0.802#	0.905* 0.905#
Chiasakul et al. (2023)	Prediction model	Integrated model^①^	249,111 inpatients	2, 3, 4, 6, 10, 12, 16	0.860 (95% CI: 0.81–0.91)	0.830 (95% CI: 0.78–0.88)	0.880* 0.850#	0.850* 0.800#
Nassour et al. (2024)	Prediction model	Random forest model	479 inpatients	1, 2, 3, 6, 10, 12, 14, 18	0.900	-	0.900*	0.760*
Guan et al. (2023)	Prediction model	Random forest model	1,647 inpatients	2, 3, 4, 6, 8, 9, 12, 16, 17	0.937	0.937	0.779* 0.779#	0.998* 0.998#
Jin et al. (2023)	Prediction model	Natural language processing	30,152 inpatients	1, 2, 3, 4, 6, 10, 12, 14, 16, 21	0.950 (95% CI: 0.94–0.96)	0.950 (95% CI: 0.94–0.96)	0.899* 0.899#	0.998* 0.998#
Franco-Moreno et al. (2023)	Prediction model	Gradient lifting decision tree	12,249 cancer patients	3, 4, 5, 6, 11, 16, 18	0.970	0.830	0.750* 0.770#	0.880* 0.930#
Hou et al. (2023)	Prediction model	Artificial neural network	801 inpatients	1, 4, 5, 6, 10, 14, 16, 17	0.970 (95% CI: 0.92–0.99)	0.960 (95% CI: 0.92–0.99)	0.920* 0.920#	0.960* 0.960#
Wu et al. (2024)	Prediction model	Random forest model	650 inpatients	2, 3, 16	0.760 (95% CI: 0.69–0.84)	0.760 (95% CI: 0.69–0.84)	0.666* 0.666#	0.876* 0.876#
Anghele et al. (2024)	Prediction model	Random forest model	299 inpatients	2, 3, 4, 6, 10, 12, 16, 18	0.990	-	0.970*	0.920*
Lin et al. (2024)	Prediction model	Gradient lifting decision tree	1,087 inpatients	2, 4, 6, 8, 10, 18	0.950	-	1.000*	0.890*
Liu et al. (2024)	Prediction model	Random forest model	620 stroke patients	1, 2, 5, 6, 12, 16	0.740	0.730	0.780* 0.800#	0.920* 0.920#
Wei et al. (2024)	Prediction model	Gradient lifting decision tree	2,434 cases of fracture patients	2, 3, 6, 10, 12, 18	0.970	-	0.956*	0.911*
Hu et al. (2025)	Prediction model	Random forest model	4,738 patients with colorectal cancer	2, 3, 4, 5, 6, 10, 11, 12, 16, 18	0.890	0.740	0.615* 0.615#	0.888* 0.740#
Liu et al. (2025)	Prediction model	Random forest model	3,116 trauma patients	2, 3, 8, 9, 10, 16	0.870 (95% CI: 0.85–0.90)	0.830 (95% CI: 0.79–0.86)	0.956* 0.821	0.687* 0.756#

① The integrated model includes gradient lifting decision tree, random forest, support vector machine and logistic regression. 1 = smoking history, 2 = age ≥70, 3 = obesity (BMI ≥30 kg/m^2^), 4 = history of venous thrombosis, 5 = decreased activity, 6 = high-risk disease factors (hypertension, coronary heart disease, diabetes, inflammatory bowel disease, varicose veins, thrombotic disease, cardiopulmonary disease, cancer, acute infection or rheumatic disease, acute myocardial infarction, ischemic stroke), 7 = hormone therapy, 8 = blood transfusion, 9 = mechanical ventilation, 10 = surgery history, 11 = chemotherapy, 12 = gender, 13 = race, 14 = anticoagulant therapy, 15 = blood glucose, 16 = laboratory indicators (D-dimer, fibrinogen, prothrombin time, international standards biochemical ratio, white blood cell count, and C-reactive protein)are included, 17 = central venous catheter (CVC) or peripherally inserted central venous catheter (PICC), 18 = length of hospital stay, 19 = diagnostic radiology, 20 = Charlson complication index, 21 = contraceptive use.

## 3 Result

### 3.1 Literature extraction

**FIGURE 2:**
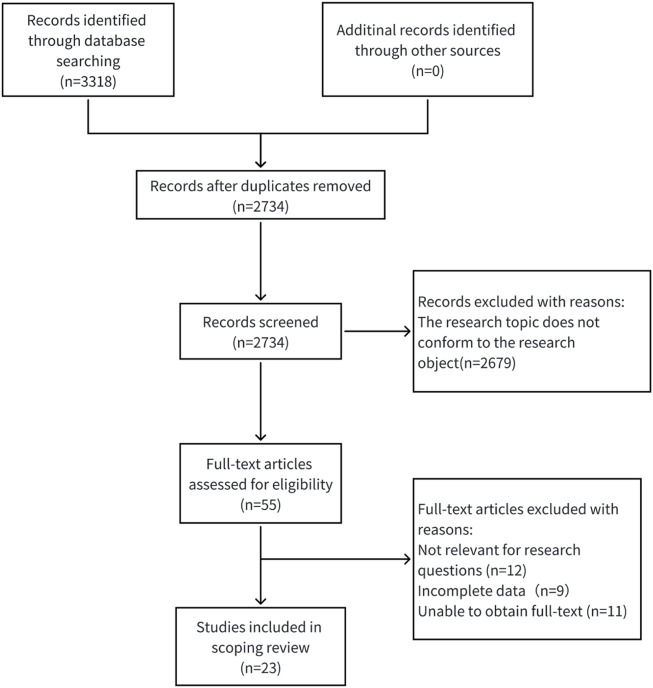
Flow chart of literature screening.

The retrieval strategy found 3,318 pieces of Chinese and English literature and their abstracts at first; after using EndNote 20.0 software to remove duplicates, 2,734 remained. Then, 2,734 were chosen by reading titles and abstracts, 55 were excluded for not meeting the criteria, 32 were removed after reading the full text, and in the end, 23 were selected (Wang et al., 2020; Nudel et al., 2021; 高远 and 李建涛, 2021; Wang et al., 2021; Liu et al., 2021; Ryan et al., 2021; Park et al., 2021; Jin et al., 2022; Li et al., 2022; Wang X. et al., 2023; Chiasakul et al., 2023; Nassour et al., 2024; Guan et al., 2023; Jin et al., 2023; Franco-Moreno et al., 2023; Hou et al., 2023; Wu et al., 2024; Anghele et al., 2024; Lin et al., 2024; Liu et al., 2024; Wei et al., 2024; Hu et al., 2025; Liu et al., 2025). The specific screening process is shown in Figure 2.

### 3.2 Basic characteristics of included literature

Among the 23 literature included in the study, 1 was in Chinese (高远 and 李建涛, 2021), and 22 were in English (Wang et al., 2020; Nudel et al., 2021; Wang et al., 2021; Liu et al., 2021; Ryan et al., 2021; Park et al., 2021; Jin et al., 2022; Li et al., 2022; Wang X. et al., 2023; Chiasakul et al., 2023; Nassour et al., 2024; Guan et al., 2023; Jin et al., 2023; Franco-Moreno et al., 2023; Hou et al., 2023; Wu et al., 2024; Anghele et al., 2024; Lin et al., 2024; Liu et al., 2024; Wei et al., 2024; Hu et al., 2025; Liu et al., 2025). The tools used for predictions include various artificial intelligence technologies, such as 10 random forest models, 5 gradient boosting decision trees, 2 natural language processing tools, 2 integrated models, 1 artificial neural network, 1 support vector machine, 1 linear discriminant analysis, and 1 generalized linear model. The subjects were hospitalized patients (*n =* 16), cancer patients (*n =* 2), orthopedic patients (*n =* 2), surgical patients (*n =* 1), stroke patients (*n =* 1), and trauma patients (*n =* 1). The main research designs were cohort study (*n =* 17), meta-analysis (*n =* 2), randomized controlled study (*n =* 2), case-control study (*n =* 1), and prospective study (*n =* 1).

### 3.3 Evaluation of risk factors and reliability and validity indicators included in the literature

**FIGURE 3:**
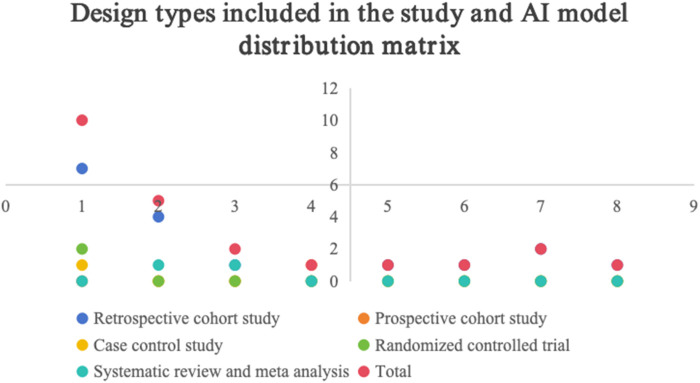
Design types and AI model distribution matrix included in the study.

**FIGURE 4:**
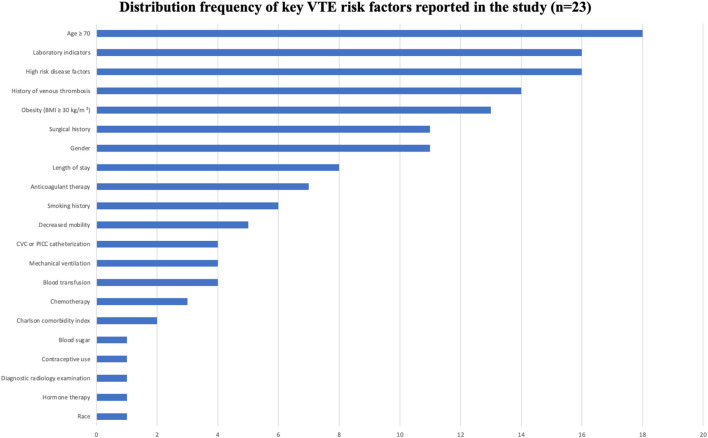
Distribution of risk factors included in the study.

See Tables 1, 2, Figures 3, 4.

**TABLE 2:** Classification of risk factors included in the literature.

Risk factor	Main assessment contents	Related literature
Patient factors	1 = Smoking history	Wang et al. (Wang et al., 2020); Nudel et al. (Nudel et al., 2021); Nassour et al. (Nassour et al., 2024); Jin et al. (Jin et al., 2023); Hou et al. (Hou et al., 2023); Liu et al. (Liu et al., 2024)
	2 = Age ≥70	Wang et al. (Wang et al., 2020); Nudel et al. (Nudel et al., 2021); Wang et al. (Wang et al., 2021); Jung et al. (Park et al., 2021); Jin et al. (Jin et al., 2022); Li et al. (Li et al., 2022); Wang et al. (Wang et al., 2023a); Chiasakul et al. (Chiasakul et al., 2023); Nassour et al. (Nassour et al., 2024); Guan et al. (Guan et al., 2023); Jin et al. (Jin et al., 2023); Wu et al. (Wu et al., 2024); Anghele et al. (Anghele et al., 2024); Lin et al. (Lin et al., 2024); Liu et al. (Liu et al., 2024); Wei et al. (Wei et al., 2024); Hu et al. (Hu et al., 2025); Liu et al. (Liu et al., 2025)
	3 = Obesity (BMI ≥30 kg/m^2^)	Wang et al. (Wang et al., 2020); Nudel et al. (Nudel et al., 2021); Wang et al. (Wang et al., 2023a); Chiasakul et al. (Chiasakul et al., 2023); Nassour et al. (Nassour et al., 2024); Guan et al. (Guan et al., 2023); Jin et al. (Jin et al., 2023); Moreno et al. (Franco-Moreno et al., 2023); Wu et al. (Wu et al., 2024); Anghele et al. (Anghele et al., 2024); Wei et al. (Wei et al., 2024); Hu et al. (Hu et al., 2025); Liu et al. (Liu et al., 2025)
	5 = Decreased mobility	Wang et al. (Wang et al., 2020); Moreno et al. (Franco-Moreno et al., 2023); Hou et al. (Hou et al., 2023); Liu et al. (Liu et al., 2024); Hu et al. (Hu et al., 2025)
	12 = Gender	Nudel et al. (Nudel et al., 2021); Wang et al. (Wang et al., 2021); Wang et al. (Wang et al., 2023a); Chiasakul et al. (Chiasakul et al., 2023); Nassour et al. (Nassour et al., 2024); Guan et al. (Guan et al., 2023); Jin et al. (Jin et al., 2023); Anghele et al. (Anghele et al., 2024); Liu et al. (Liu et al., 2024); Wei et al. (Wei et al., 2024); Hu et al. (Hu et al., 2025)
	13 = Race	Nudel et al. (Nudel et al., 2021)
Disease factors	4 = History of venous thrombosis	Wang et al. (Wang et al., 2020); Gao et al. (高远 and 李建涛, 2021); Liu et al. (Liu et al., 2021); Ryan et al. (Ryan et al., 2021); Jin et al. (Jin et al., 2022); Wang et al. (Wang et al., 2023a); Chiasakul et al. (Chiasakul et al., 2023); Guan et al. (Guan et al., 2023); Jin et al. (Jin et al., 2023); Moreno et al. (Franco-Moreno et al., 2023); Hou et al. (Hou et al., 2023); Anghele et al. (Anghele et al., 2024); Lin et al. (Lin et al., 2024); Hu et al. (Hu et al., 2025)
	6 = High risk disease factors	Wang et al. (Wang et al., 2020); Nudel et al. (Nudel et al., 2021); Ryan et al. (Ryan et al., 2021); Jung et al. (Park et al., 2021); Wang et al. (Wang et al., 2023a); Chiasakul et al. (Chiasakul et al., 2023); Nassour et al. (Nassour et al., 2024); Guan et al. (Guan et al., 2023); Jin et al. (Jin et al., 2023); Moreno et al. (Franco-Moreno et al., 2023); Hou et al. (Hou et al., 2023); Anghele et al. (Anghele et al., 2024); Lin et al. (Lin et al., 2024); Liu et al. (Liu et al., 2024); Wei et al. (Wei et al., 2024); Hu et al. (Hu et al., 2025)
	18 = Length of stay	Jung et al. (Park et al., 2021); Jin et al. (Jin et al., 2022); Nassour et al. (Nassour et al., 2024); Moreno et al. (Franco-Moreno et al., 2023); Anghele et al. (Anghele et al., 2024); Lin et al. (Lin et al., 2024); Wei et al. (Wei et al., 2024); Hu et al. (Hu et al., 2025)
	20 = Charlson comorbidity index	Jung et al. (Park et al., 2021); Jin et al. (Jin et al., 2022)
Therapeutic factors	7 = Hormone therapy	Wang et al. (Wang et al., 2020)
	8 = Blood transfusion	Wang et al. (Wang et al., 2020); Guan et al. (Guan et al., 2023); Lin et al. (Lin et al., 2024); Liu et al. (Liu et al., 2025)
	9 = Mechanical ventilation	Wang et al. (Wang et al., 2020); Guan et al. (Guan et al., 2023); Liu et al. (Liu et al., 2025)
	10 = Surgical history	Wang et al. (Wang et al., 2020); Jung et al. (Park et al., 2021); Wang et al. (Wang et al., 2023a); Chiasakul et al. (Chiasakul et al., 2023); Nassour et al. (Nassour et al., 2024); Jin et al. (Jin et al., 2023); Hou et al. (Hou et al., 2023); Anghele et al. (Anghele et al., 2024); Wei et al. (Wei et al., 2024); Hu et al. (Hu et al., 2025); Liu et al. (Liu et al., 2025)
	11 = Chemotherapy	Wang et al. (Wang et al., 2020); Moreno et al. (Franco-Moreno et al., 2023); Hu et al. (Hu et al., 2025)
	14 = Anticoagulant therapy	Nudel et al. (Nudel et al., 2021); Gao et al. (高远 and 李建涛, 2021); Jung et al. (Park et al., 2021); Wang et al. (Wang et al., 2023a); Nassour et al. (Nassour et al., 2024); Jin et al. (Jin et al., 2023); Hou et al. (Hou et al., 2023)
	19 = Diagnostic radiology examination	Jung et al. (Park et al., 2021)
	21 = Contraceptive use	Jin et al. (Jin et al., 2023)
Clinical index factors	15 = Blood sugar	Gao et al. (高远 and 李建涛, 2021)
	16 = Laboratory indicators	Gao et al. (高远 and 李建涛, 2021); Wang et al. (Wang et al., 2021); Liu et al. (Liu et al., 2021); Jin et al. (Jin et al., 2022); Li et al. (Li et al., 2022); Wang et al. (Wang et al., 2023a); Chiasakul et al. (Chiasakul et al., 2023); Guan et al. (Guan et al., 2023); Jin et al. (Jin et al., 2023); Moreno et al. (Franco-Moreno et al., 2023); Hou et al. (Hou et al., 2023); Wu et al. (Wu et al., 2024); Anghele et al. (Anghele et al., 2024); Liu et al. (Liu et al., 2024); Hu et al. (Hu et al., 2025); Liu et al. (Liu et al., 2025)
Catheter factor	17 = CVC or PICC catheterization	Liu et al. (Liu et al., 2021); Ryan et al. (Ryan et al., 2021); Guan et al. (Guan et al., 2023); Hou et al. (Hou et al., 2023)

## 4 Discussion

### 4.1 There are various types of risk assessment models of artificial intelligence in venous thrombosis, but most models lack external validation and critical appraisal

At present, the research on AI technology for evaluating VTE with AI models is gradually increasing, including random forests model, gradient lifting decision trees, natural language processing, integrated model, artificial neural network, support vector machine model, linear discriminant analysis, and generalized linear models. Among the 23 studies included in this study, the predictive performance was evaluated, but 7 studies were not externally verified. The AUC of the test set ranged from 0.773 to 0.990 (高远 and 李建涛, 2021; Park et al., 2021; Jin et al., 2022; Nassour et al., 2024; Anghele et al., 2024; Lin et al., 2024; Wei et al., 2024). At present, relevant studies have externally verified some prediction tools to determine the clinical applicability of AI prediction model. Among the 23 prediction models, 16 models were developed and validated. The AUC range of the test set was 0.740–0.970, and the AUC range of the validation set was 0.740–0.980 (Wang et al., 2020; Nudel et al., 2021; Wang et al., 2021; Liu et al., 2021; Ryan et al., 2021; Li et al., 2022; Wang X. et al., 2023; Chiasakul et al., 2023; Guan et al., 2023; Jin et al., 2023; Franco-Moreno et al., 2023; Hou et al., 2023; Wu et al., 2024; Liu et al., 2024; Hu et al., 2025; Liu et al., 2025).

### 4.2 Technical characteristics, application variations, and clinical translation challenges of AI models

Different AI methods exhibit distinct characteristic differences and applicable scenarios when handling VTE risk prediction. Tree-based models such as random forests and gradient boosting decision trees excel in capturing complex nonlinear relationships and feature interactions, making them particularly suitable for high-dimensional clinical data; however, they suffer from poor model interpretability. All ten studies employing random forests in this research reported high predictive performance (AUC >0.85), yet their “black-box” nature limits clinicians’ understanding and trust in the predictions. Natural language processing (NLP) techniques can extract key information from unstructured electronic medical record text. For example, Wang et al. (2021) achieved an AUC of 0.970 using an NLP model, highlighting its unique advantage in mining the value of clinical notes, although its performance is highly dependent on corpus quality and annotation consistency. Linear models such as support vector machines (SVM) and linear discriminant analysis (LDA), while offering strong interpretability and high computational efficiency, lack the flexibility to recognize complex data patterns. For instance, the SVM model adopted by Liu et al. (2021) achieved an AUC of 0.904 but required extensive feature engineering.

It is noteworthy that significant differences also exist among AI methods in feature selection and model generalizability. Tree models typically possess built-in feature importance evaluation functions, enabling automatic identification of key risk factors, but are prone to overfitting the training data. Although ensemble models improve predictive stability by combining multiple base learners, this comes at the cost of increased model complexity and computational resource demands. Furthermore, existing studies generally lack validation of models’ cross-institutional generalizability. Only a few studies (Nudel et al., 2021; Ryan et al., 2021; Guan et al., 2023) used multi-center data for external validation, while data standardization issues (such as differences in laboratory measure units, inconsistent diagnostic codes, etc.) remain critical obstacles affecting model transferability.

At the level of clinical feasibility and practical implementation, the application of AI models faces numerous challenges. Firstly, insufficient model interpretability affects clinical adoption, as physicians struggle to understand the decision-making basis of “black-box” models. Secondly, real-time prediction requires deep integration with existing EMR systems, involving a series of practical issues such as data interfaces, computational efficiency, and modifications to user workflows. Additionally, no consensus framework has yet been formed for the regulatory approval and ethical considerations of AI models in clinical practice.

Therefore, future research should not solely focus on improving model predictive accuracy but should pay more attention to the clinical translation pathway of AI models. It is recommended to prioritize the development of highly interpretable models that can seamlessly integrate into clinical decision-making workflows. Simultaneously, multi-center collaboration should be strengthened to establish standardized VTE data collection and model validation protocols, and to actively explore model deployment and regulatory solutions that comply with healthcare industry standards.

### 4.3 Analysis of risk factors of artificial intelligence in venous thrombosis

AI models integrated multi-dimensional risk factors, encompassing five major categories: patient factors, disease factors, treatment factors, laboratory indicators, and catheter-related factors. Factors that appeared frequently (reported in ≥10 studies) included: age ≥70 years, obesity (BMI ≥30 kg/m^2^), gender, history of VTE, high-risk comorbidities, surgical history, and abnormal laboratory indicators. These factors were consistently identified as important predictive variables across multiple models, demonstrating strong evidence consistency.

#### 4.3.1 Comprehensive assessment of patient-related factors is fundamental for reducing the risk of venous thrombosis

① Smoking significantly increases the risk of VTE through mechanisms involving vascular endothelial injury, activation of inflammatory responses, and a hypercoagulable state (Wang et al., 2020; Catella et al., 2022). The AI model developed by De Pooter et al. (2021) demonstrated that a history of smoking significantly enhanced the predictive accuracy for VTE (AUC = 0.856), while Nudel et al. (2021) found that smokers faced a 50%–100% higher risk of VTE compared to non-smokers. These findings suggest that clinical practice should more systematically evaluate the impact of smoking on VTE, particularly in high-risk populations, by strengthening smoking cessation interventions to reduce the disease burden associated with smoking-related VTE. ② Age ≥70 years is an independent risk factor for VTE, with its mechanism linked to declining vascular endothelial function and venous stasis (Righini et al., 2014). The random forest model by Wang et al. (2021) indicated a significant weight for this factor (AUC = 0.970), and Park et al. (2021) observed that the incidence of VTE in elderly patients was 2–3 times higher than in younger populations. There is a clinical need to implement intensified VTE prevention strategies for elderly patients, and future research could explore AI-driven risk stratification models that incorporate aging-related biomarkers. ③ Obesity (BMI ≥30 kg/m^2^) elevates VTE risk by promoting a chronic inflammatory state, hypercoagulability, and hemodynamic changes (Chen et al., 2022; Goffi et al., 2025). The study by Gao et al. (高远 and 李建涛, 2021) showed that obesity increased the risk of VTE by 2.5 times (AUC = 0.890), and Nudel et al. (2021) identified BMI ≥30 kg/m^2^ as a core predictive factor in their bariatric surgery cohort. Clinicians should pay special attention to VTE prevention in obese patients, applying mechanical and pharmacological prophylactic measures appropriately. ④ Decreased mobility leads to impaired muscle pump function and venous stasis, significantly increasing the risk of VTE (Chatterjee et al., 2021; Nayak et al., 2024). A study by Wang et al. (2020) showed that individuals with restricted mobility had a 3.2-fold higher risk of VTE, while Franco-Moreno et al. (2023) demonstrated a dose-dependent relationship between bed rest duration and DVT incidence (OR = 1.8, 95% CI 1.2–2.7). Therefore, early mobility interventions should be emphasized, and optimal activity protocols for different patient populations need to be explored. ⑤ Gender differences play a significant role in VTE risk. Women of reproductive age face elevated risk due to estrogen effects, while older men exhibit high risk due to the accumulation of risk factors (Schapkaitz et al., 2023; Giustozzi et al., 2021). Nudel et al. (2021) identified male sex as an independent predictor (OR = 1.4), and Wang et al. (2021) incorporated gender as a core variable in their natural language processing model (AUC = 0.970). Clinical risk assessment should account for gender differences, and future efforts should focus on developing gender-specific prediction models. ⑥ Racial disparities are associated with genetic predisposition, inflammatory status, and medical interventions. Although African populations have a lower prevalence of inherited thrombophilia, they exhibit a higher risk of VTE (Saber et al., 2022). Nudel et al. (2021) found that African American patients had a 1.3-fold increased risk (OR = 1.3), and Wang X. et al. (2023) validated race as an independent predictive variable using an integrated model (AUC = 0.920). Future research should further explore gene-environment interactions related to race and uncover underlying biological mechanisms through the integration of multi-omics data and AI modeling.

#### 4.3.2 Effective management of high-risk diseases is central to reducing the risk of venous thrombosis

① A history of venous thromboembolism (VTE) is the strongest independent risk factor for VTE recurrence. The mechanisms involve a persistent hypercoagulable state, abnormal repair following vascular endothelial injury, and the procoagulant effect of residual thrombosis (Simon et al., 2023). A study by Gao et al. (高远 and 李建涛, 2021) in orthopedics showed that this history increases VTE risk by 4.8-fold (AUC = 0.890), while Wang X. et al. (2023) identified it as the highest-weight predictor in their integrated model (AUC = 0.920). Consequently, long-term risk management—including extended anticoagulation therapy and regular monitoring of relevant indicators—is essential for patients with a prior VTE event (Simon et al., 2023). ② High-risk diseases synergistically increase VTE risk through multiple mechanisms: metabolic diseases cause endothelial glycation injury, cardiovascular disorders lead to abnormal blood flow, inflammatory conditions activate coagulation factors via cytokines, and malignancies directly release procoagulant substances (Charlier et al., 2022; Egbe et al., 2018; Patiño-Trives et al., 2021). Wang et al. (2021) confirmed that the presence of ≥2 high-risk diseases elevates VTE risk by 4.3 times (AUC = 0.856), a correlation further validated by Chiasakul et al. (2023) and Wei et al. (2024). In clinical practice, it is crucial to establish disease-specific risk assessment systems and develop targeted prevention strategies for specific disease combinations to optimize individualized VTE management in high-risk patients. ③ Prolonged hospital stay is significantly associated with increased VTE risk, which is attributed to reduced mobility, impaired muscle pump function, and venous stasis in the lower limbs (Tøndel et al., 2022). Park et al. (2021) found that for every 5-day increase in hospitalization, VTE risk rises by 1.8-fold—a conclusion supported by Nassour et al. (2024) (AUC = 0.900) and Liu et al. (2025) (OR = 2.1). Future research should focus on developing dynamic risk assessment systems based on real-time monitoring data and integrating them with electronic health records to enable automated alerts. ④ The Charlson Comorbidity Index (CCI), which reflects comorbidity burden, shows a significant correlation with VTE risk. The underlying mechanisms involve systemic inflammation, activation of a procoagulant state, and the impact of polypharmacy (Bonnesen et al., 2022). Park et al. (2021) demonstrated that patients with a CCI ≥3 have a 3.5-fold higher VTE risk, and Jin et al. (2022) included CCI as a core predictor (AUC = 0.773). Therefore, greater emphasis should be placed on thromboprophylaxis in patients with comorbidities, especially high-risk individuals with CCI ≥2. Future efforts may explore comorbidity-specific risk prediction models and develop individualized prevention and treatment strategies for this special population.

#### 4.3.3 Making reasonable treatment decisions is a crucial measure for reducing the risk of venous thrombosis

① Hormone therapy is unequivocally associated with an increased risk of VTE, primarily due to disruption of the coagulation balance. Exogenous hormones promote the synthesis of clotting factors and inhibit anticoagulant proteins, while glucocorticoids suppress the fibrinolytic system (Ayodele et al., 2022). Wang et al. (2020) reported that hormone therapy increases VTE risk by 2.8-fold, and Jin et al. (2023) confirmed it as an independent predictor in women of reproductive age using an NLP model (AUC = 0.950). Individualized thrombotic risk assessment should be conducted prior to initiating hormone therapy to optimize safety in patients requiring long-term hormonal treatment. ② Blood transfusion is associated with an elevated risk of VTE. Microparticles and free hemoglobin in stored blood trigger inflammation and endothelial activation, while also enhancing platelet activity and thrombin generation (Sheth et al., 2022). Wang et al. (2020) found that perioperative transfusion increased VTE risk by 3.2-fold, and Guan et al. (2023) supported transfusion as an independent predictor using a random forest model (AUC = 0.937). These findings underscore the importance of strict adherence to transfusion indications and enhanced thrombotic monitoring in patients requiring transfusion. ③ Mechanical ventilation significantly increases VTE risk. Positive pressure ventilation and sedation-induced immobility reduce venous return and impair muscle pump function (Sochet et al., 2022). Wang et al. (2020) observed a VTE incidence of 28.6% in mechanically ventilated patients, and Liu et al. (2025) indicated an 18% increase in risk for every additional 24 h of ventilation. Future research should focus on developing dynamic risk assessment models based on ventilation parameters and evaluating the impact of alternative therapies, such as extracorporeal support, on thromboprophylaxis to optimize VTE management in critically ill patients. ④ Surgical history is an independent risk factor for VTE. Tissue injury during the perioperative period releases tissue factor that activates coagulation, while immobility contributes to venous stasis (Levy et al., 2023). Anghele et al. (2024) reported a 5.8-fold increase in VTE risk following major surgery, and Wang X. et al. (2023) identified it as the third most important predictor in their integrated model. Therefore, healthcare providers should implement stratified prophylaxis strategies for surgical patients, selecting appropriate anticoagulation regimens based on surgery type and individual patient characteristics.⑤ Chemotherapy significantly increases the risk of VTE through direct endothelial injury, promotion of tissue factor release, and suppression of anticoagulant proteins (Izquierdo-Condoy et al., 2025). Franco-Moreno et al. (2023) reported a VTE incidence of 12.8% in chemotherapy patients, while Hu et al. (2025) found that regimens containing bevacizumab were associated with an additional 2.1-fold increase in risk. Future efforts should focus on developing chemotherapy cycle-specific dynamic prediction models that integrate pharmacogenomic profiles and real-time biomarker monitoring to optimize thromboprophylaxis strategies in cancer patients. ⑥ Anticoagulant therapy exhibits a “biphasic effect” on VTE risk: an initial transient hypercoagulable state may occur, while long-term suboptimal adherence or inadequate dosing increases the risk of recurrence (Li et al., 2023). Nudel et al. (2021) demonstrated a 2.3-fold higher recurrence risk in patients with subtherapeutic anticoagulation, and Hou et al. (2023) validated anticoagulation intensity as a key predictive variable using a neural network model (AUC = 0.970). Clinical practice should emphasize drug monitoring and patient education, alongside the development of intelligent dose-adjustment systems based on real-time coagulation monitoring to enable precision thrombosis management. ⑦ Diagnostic radiological procedures increase VTE risk due to prolonged immobility in specific positions and the use of contrast agents, which can cause endothelial injury and coagulation activation (Oka et al., 2020). Park et al. (2021) observed a significant rise in VTE risk within 30 days after contrast-enhanced CT (OR = 2.1), particularly among bedridden patients. Future research should explore methods to mitigate contrast-induced endothelial damage and develop risk assessment tools tailored to examination type and duration. ⑧ Contraceptive use significantly elevates VTE risk, as exogenous estrogen upregulates the synthesis of clotting factors and suppresses anticoagulant protein activity (Maughan et al., 2022; Gialeraki et al., 2018). Jin et al. (2023) reported a 3.5-fold increased risk among users, with even higher risk (OR = 6.8) in carriers of Factor V Leiden mutation. Contraceptive prescriptions should be carefully considered and preceded by individualized risk assessment, especially in patients with inherited thrombophilia or other risk factors.

#### 4.3.4 Dynamic monitoring of clinical indicators is essential for reducing the risk of venous thrombosis

① Hyperglycemia exhibits a dose-response relationship with VTE risk, primarily mediated through endothelial dysfunction, platelet activation, and impaired fibrinolysis (Niu et al., 2019). Gao et al. (高远 and 李建涛, 2021) demonstrated that fasting blood glucose >7.0 mmol/L increased VTE risk by 2.3-fold, with random forest models identifying glucose as a core predictive variable (AUC = 0.890). Diabetic patients face particularly elevated risk due to chronic inflammation and hemorheological alterations. These findings underscore the importance of enhanced glycemic monitoring and thromboprophylaxis in patients with hyperglycemia. ② Abnormal laboratory indicators reflect multidimensional imbalances in coagulation, inflammation, and hemorheology. Elevated D-dimer indicates coagulation activation; increased fibrinogen promotes thrombus formation and exacerbates stasis; abnormal clotting times reflect dysregulated coagulation factors; and inflammatory markers such as C-reactive protein contribute to thrombosis via procoagulant mechanisms (Wang J. et al., 2023; Sleutjes et al., 2021). Li et al. (2022) confirmed that combining D-dimer (>0.5 mg/L) and fibrinogen (>4 g/L) improved predictive performance (AUC = 0.839), while Wang et al. (2021) found that dynamic changes in these markers offered greater value than single measurements (AUC = 0.970). Future research should focus on developing machine learning models capable of integrating temporal variations in multiple biomarkers and exploring the predictive utility of novel biomarkers to enable earlier risk warning and precision interventions.

#### 4.3.5 Routine catheter care is imperative for reducing reduce the risk of venous thrombosis

The presence of a central venous catheter (CVC) or peripherally inserted central catheter (PICC) significantly increases the risk of venous thromboembolism (VTE). The underlying mechanisms include vascular injury, altered hemodynamics, and catheter-blood interface interactions. Catheter insertion causes direct vascular endothelial damage and activates the coagulation cascade. The persistent presence of the catheter alters blood flow patterns, creating turbulence and low-shear zones that promote platelet adhesion (Citla Sridhar et al., 2020; Lockwood and Desai, 2019). A prospective study by Liu et al. (2021) reported a symptomatic VTE incidence of 15.3% in patients with CVC/PICC (OR = 4.2), and Ryan et al. (2021) identified catheter-related factors as an independent predictor using a gradient-boosted decision tree model (AUC = 0.830). The risk of catheter-related thrombosis is closely associated with insertion site, catheter diameter, and indwelling duration. Clinical practice should adhere to best practices in catheter management, including ultrasound-guided insertion, minimizing catheter size, and regularly reassessing the necessity of catheter retention. Future efforts should explore personalized catheterization strategies based on individual vascular anatomy and develop predictive models integrating clinical factors and biomarkers to enable early warning and precision prevention of catheter-related thrombosis.

### 4.4 Sources and evidence synthesis of heterogeneity in AI model performance

The AI models synthesized in this study demonstrated excellent predictive performance (AUC: 0.740–0.990), yet significant heterogeneity was observed in their outcomes. This variation is not coincidental but stems primarily from three sources. First, differences at the data level play a central role. The included studies utilized diverse data sources—some derived from single-center electronic health records, while others originated from multi-center databases or disease-specific registries. Variations in data quality, completeness, and coding consistency directly influence model performance. For example, studies by Wang et al. (2021) and Jin et al. (2023), which leveraged large, rigorously validated data warehouses, achieved notably high AUC values above 0.95. Second, considerable disparities in sample sizes and the number of outcome events contributed to differences in model stability. Studies with sample sizes exceeding ten thousand cases (Vollmer et al., 2020) generally demonstrated better model generalizability compared to those with smaller samples (Nudel et al., 2021), even though the latter occasionally reported very high AUC values at the risk of overfitting. Finally, variations in model algorithms and validation methods introduced additional heterogeneity. Although ensemble learning models overall performed superiorly, their efficacy heavily depended on hyperparameter tuning and the rigor of internal validation strategies.

Therefore, when interpreting the predictive performance of these AI models, it is essential to critically consider the design context and data foundations of the original studies. Future research should focus on establishing standardized data reporting protocols and model validation workflows to facilitate evidence integration and comparison across the field.

## 5 Limitations

Based on the analysis of the 23 included studies, 70% (*n =* 16) were retrospective in design, which may introduce recall bias. Furthermore, while this study focuses on predictive performance, the “black-box” nature of most AI models remains a significant challenge in terms of interpretability. Although these models can effectively predict risk, they often fail to provide clinicians with intuitive decision-making rationale, thereby hindering clinical translation. Finally, as a scoping review, this study aims to outline the overall landscape of the field but does not include meta-analysis of model performance, which limits the ability to draw definitive conclusions regarding the effectiveness of the models. Future research should incorporate more prospective designs, strive to develop interpretable AI models, and explore validation of model generalizability across different studies and populations.

## 6 Conclusion

This paper systematically reviews the application status of AI in the VTE risk assessment through a scoping review. The study found that AI models such as random forest, gradient boosting decision tree, and natural language processing demonstrated high performance in predicting VTE risk, with AUC values between 0.740 and 0.990, which was much better than traditional risk assessment scores. In addition, AI can integrate multi-source data, including patient factors, disease factors, treatment factors, laboratory indicators, and catheter factors, to achieve dynamic and personalized risk assessment. Among them, age ≥70 years old, obesity, history of venous thrombosis, and abnormal laboratory indicators were identified as key predictive factors. It provides a more accurate risk stratification tool for clinical practice and helps optimize VTE prevention and treatment strategies. Future studies should further explore multicenter, prospective data to verify the universality and clinical practicability of AI models. At the same time, the development of a dynamic prediction model that can integrate real-time monitoring data and realize automatic early warning combined with an electronic medical record system will greatly improve the efficiency of VTE management. In addition, in the future, we can pay attention to the personalized model of specific populations and explore the combination of new biomarkers and AI technology, which is expected to open up a new way for the prevention and treatment of VTE. Through the above ways, AI technology is continuously optimized to achieve early intervention and precise management of VTE, reduce the disease burden, and improve the prognosis of patients.
